# Effect of Treatment with Colchicine after Acute Coronary Syndrome on Major Cardiovascular Events: A Systematic Review and Meta-Analysis of Clinical Trials

**DOI:** 10.1155/2022/8317011

**Published:** 2022-04-13

**Authors:** Erfan Razavi, Akam Ramezani, Asma Kazemi, Armin Attar

**Affiliations:** ^1^Students' Scientific Research Center, Tehran University of Medical Sciences, Tehran, Iran; ^2^Nutrition Research Center, Shiraz University of Medical Sciences, Shiraz, Iran; ^3^Department of Cardiovascular Medicine, TAHA Clinical Trial Group, School of Medicine, Shiraz University of Medical Sciences, Shiraz, Iran

## Abstract

**Aim:**

Colchicine as an anti-inflammatory drug might be effective in the treatment of atherosclerosis, an inflammatory-based condition. The aim of this systematic review and meta-analysis was to evaluate the impact of colchicine on acute coronary syndrome (ACS).

**Methods:**

We searched SCOPUS, PubMed, and Web of Science up to September 27, 2020. All clinical trials which evaluated the effect of colchicine on ACS patients and reported high-sensitivity C-reactive protein (hs-CRP) serum level or gastrointestinal (GI) adverse events with at least 5-day follow-up or death, myocardial infarction (MI), and stroke with at least 30-day follow-up as outcomes were included.

**Results:**

Finally, seven publications were analyzed. The results of our study revealed that colchicine has a marginally significant effect on hs-CRP attenuation. Furthermore, colchicine manifested promising results by declining the risk of stroke by 70%. However, MI and primary composite endpoint did not differ between the colchicine and noncolchicine groups. Although colchicine did not significantly increase GI adverse events in the pooled analysis, the dose-dependent effect was detected. Low-dose consumption can avoid GI side effects of colchicine.

**Conclusion:**

Colchicine has shown some molecular and clinical promising results in ACS patients. The lack of effect of colchicine on MI and all-cause mortality can be partly attributed to the limitations of previous studies. Since colchicine is an inexpensive and easy-to-access drug that has shown to be safe in low-dose regimens in the clinical setting; it would be worthy that future large-scale well-designed clinical trials address this issue by resolving the limitations of previous investigations.

## 1. Introduction

Inflammation plays a prominent role in the pathophysiology of atherosclerosis [1]. Previous studies indicated the correlation between inflammatory response and infarct size. Moreover, adverse events after acute myocardial infarction (AMI) were found to be associated with inflammatory response [2]. Several therapeutic approaches with different efficacy have been developed to address anti-inflammatory needs after acute coronary syndrome such as canakinumab or colchicine with anti-inflammatory mechanism of action [3]. Canakinumab, for instance, is a recombinant human monoclonal antibody targeting interleukin-1*β* that has anti-inflammatory and plaque modification effects in patients with atherosclerotic disease. A previous study revealed that a 150 mg dose of canakinumab every 3 months resulted in a significantly lower risk of recurrent cardiovascular events in patients with previous myocardial infarction (MI) when compared to placebo. However, a higher incidence of fatal infections was reported in the intervention group [4]. The promising effect of canakinumab on cardiovascular events encouraged the researchers to examine other anti-inflammatory drugs like colchicine in order to both reach similar results and resolve the problems of canakinumab.

Colchicine, an ancient anti-inflammatory medicine, has been employed for the treatment and prevention of diseases like gout, familial Mediterranean fever (FMF), Behçet's syndrome, and many other inflammatory disorders [5–9]. Colchicine's approval by Food and Drug Administration (FDA) in 2009 [10] attracted attention, and since then, it has been widely studied as a possible therapy for cardiovascular diseases [11, 12]. Colchicine has been proven to be useful in the primary and secondary prevention of pericarditis [13]. A cross-sectional study also indicated that using colchicine in gout patients diminished the prevalence of MI along with a likely promising impact on other complications of disease [14].

As mentioned previously, reduction of inflammation with anti-inflammatory drugs such as colchicine has emerged as a therapeutic option for secondary prevention in coronary artery disease (CAD) [15]. The level of high-sensitivity C-reactive protein (hs-CRP) is elevated in nearly 60% of patients with ACS [16]. CRP is a strong independent predictor for secondary cardiovascular outcomes after ACS [17]. Colchicine can rapidly reduce the level of inflammation biomarkers, specially hs-CRP [18, 19]. Thus, a reduction in the risk for cardiovascular events is expected after taking colchicine.

Thus, colchicine could be an inexpensive logical medication for ACS patients. Recently, results from two large randomized trials in the field have been published. While in the Colchicine Cardiovascular Outcomes Trial (COLCOT), colchicine showed promising benefits for secondary prevention of major cardiovascular events (MACE) [12], in the Australian COPS trial, contrary results were observed [20]. Consequently, it is necessary to conduct a meta-analysis to explore the effect of this medication on reducing MACE after ACS. There are several systematic reviews on this topic, but none of them have distinguished between stable coronary artery disease and acute coronary syndrome, while no meta-analysis existed considering this point. These two should be treated as distinct entities due to different pathophysiology in some aspects. Therefore, there is a need for a systematic review and meta-analysis with a specific focus on ACS. The aim of this meta-analysis is to determine the effect of colchicine on the prevention of cardiovascular events after ACS and to explore its adverse events.

## 2. Methods

### 2.1. Search Strategy

This study was designed according to the guidelines of the 2009 preferred reporting items for systematic reviews and meta-analysis (PRISMA) statement [21]. The PICOS (participants, intervention, comparison, outcome, study design) model was used to formulate the study question (Table 1). SCOPUS, PubMed, and Web of Science databases were searched using the following search terms in titles and abstracts (also in combination with MESH terms): (Colchicine) AND (“Myocardial Infarction” OR “Myocardial Infarct∗” OR MI OR STEMI OR NSTEMI OR (Infarct∗ AND Myocardial) OR “Cardiovascular Stroke∗” OR “Heart Attack∗” OR (Angina AND Unstable) OR “Unstable Angina∗” OR “Angina at Rest” OR (Angina∗ AND Preinfarction) OR “Preinfarction Angina∗” OR “Myocardial Preinfarction Syndrome∗” OR “Acute Coronary Syndrome”). The wildcard term “∗” was used to increase the sensitivity of the search strategy. The language of articles was restricted to English in the literature search. The search was limited to studies on humans. The literature was searched from inception to September 27, 2020.

### 2.2. Inclusion Criteria

All clinical trials and their reports were included if they had (1) administered colchicine as secondary prevention in ACS or MI patients and reported at least one of the following as an outcome: hs-CRP serum level or gastrointestinal (GI) adverse events with minimum 5-day follow-up or each of death, stroke, and MI recurrence with minimum 30-day follow-up.

### 2.3. Exclusion Criteria

The following are the exclusion criteria: articles lacking a description of the subjects, studies from which raw data cannot be extracted, articles without a control group, articles featuring patients with other cardiovascular diseases, repeated data publications, and papers that were not available in the full text were excluded.

### 2.4. Study Selection

Two reviewers (E.R. and A.R.) independently evaluated the eligibility of the studies. First, titles and abstracts of the retrieved studies were screened to select the articles for full-text review. Later, based on the full-text reading of the selected articles, two readers independently decided whether or not to include a specific study for data extraction. Discrepancies were resolved by discussion or consulting a third party. We contacted the corresponding author of any article which we were unable to get access to the full text or the ones in which some required data were lacking.

### 2.5. Data Extraction

Eligible data were extracted and included in a uniform data entry form, which featured publication year, title, first author's name, study location, study subjects, study design, dose, duration of colchicine therapy, duration of follow-up, number of participants in the colchicine and control groups, age, gender, hs-CRP levels before and after the intervention, number of deaths, and number of cardiovascular events. The primary composite endpoint consisted of two components: death and MI. In order to involve more studies and patients in the assessment of the impact of colchicine on the composite endpoint, other cardiovascular-related outcomes such as stroke were not counted as the components of the primary composite endpoint as were reported by few studies.

Two authors extracted data independently (E.R. and A.R.). Any dispute was settled by discussion or by a third investigator.

### 2.6. Evaluation of Literature Quality

The quality of the studies was evaluated independently by two authors (ER and AR) using the Cochrane Risk of Bias Tool for Randomized Controlled Trials. The items used for the assessment of each study were as follows: random sequence generation, allocation concealment, blinding of participants and personnel, blinding of outcome assessment, incomplete outcome data, and selective outcome reporting.

According to the recommendations of the Cochrane Handbook, a judgment of “low” indicated a low risk of bias, while “high” indicated a high risk of bias. Labeling an item as “unclear” indicated an unclear or unknown risk of bias.

### 2.7. Statistical Analysis

For hs-CRP, the mean change from baseline and its standard deviation (SD) were extracted. For two studies, only the final values were reported (ref), so for these, the final values were pooled with the other studies. The weighted mean difference (WMD) and its corresponding SD were calculated using the DerSimonian and Laird method (DerSimonian and Laird 1986) which takes the between-study variation into account. Between studies, heterogeneity was assessed using the Cochran's *Q* test and *I*^2^.

For the other outcomes (MI, death, stroke, composite endpoint, and GI adverse events), we calculated the risk ratio (RR). If at least 10 studies were available, we explored potential small-study effects, such as publication bias, using visual examination of funnel plot and Egger's test.

To find the possible sources of heterogeneity, subgroup analysis was used. Subgroup analysis was performed for colchicine dosages and types of disease where possible (≥2 studies in each subgroup). To ensure that one large study or a study with an extreme result have not influenced the results, we conducted sensitivity analyses by excluding one study at a time and reestimating the effect sizes. All the statistical procedures were performed using Stata software version 13 (StataCorp LP, College Station, TX, USA).

## 3. Results

**Figure 1:**
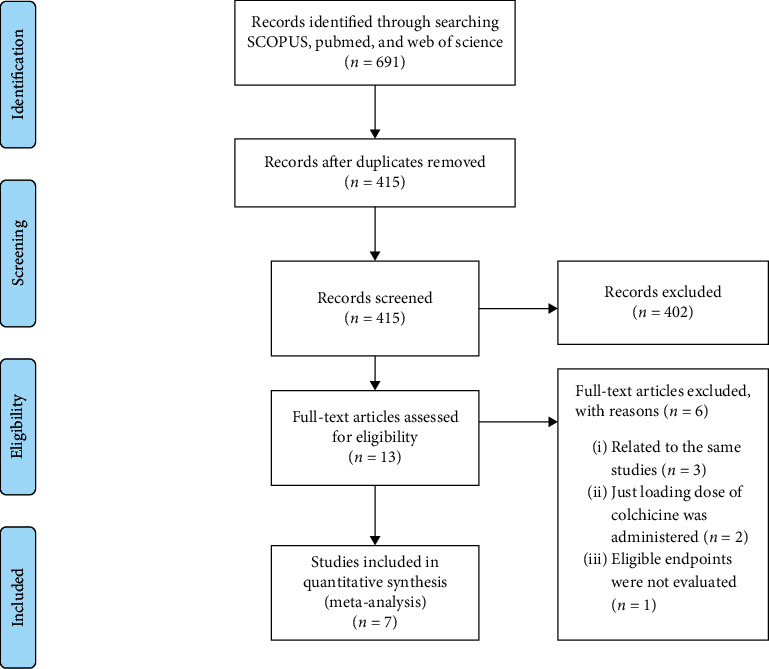
PRISMA flow diagram of study selection.

The search strategy yielded 691 articles of which 13 met the eligibility criteria, and their full-text were assessed. Of these, two studies administered colchicine once or twice prior to percutaneous coronary intervention (PCI) and did not continue the administration in the consecutive days following PCI. Moreover, 5 studies were excluded since they were published several times while pertaining to the same populations and studies. We could not get access to one article despite our endeavor to contact the corresponding author of the given article three times. Finally, seven articles were used for analysis. The PRISMA flow diagram is shown in Figure 1.

Detailed characteristics of the studies are presented in Table 2. All included studies were parallel designed. Five studies enrolled MI patients as participants, 1 study enrolled ACS patients, and the other one evaluated ACS or ischemic stroke patients. The trial duration ranged from 5 days to 22.6 months. The number of participants in each study ranged from 32 to 4745. The mean age of the colchicine or control groups ranged from 52.8 to 62.1 years. Colchicine dosage varied across different studies: three studies administered 0.5 mg once a day, 2 studies administered 1 mg once a day, 1 study administered 0.5 mg twice a day for the first month and then 0.5 mg once a day for the following eleven months, and another study used 2 mg as a loading dose plus 0.5 mg colchicine daily. Six studies were double-blind, while one study was nonblind. Therefore, we conducted sensitivity analysis by excluding this study. The risk of bias in the studies is presented in Table 3.

### 3.1. hs-CRP

**Figure 2:**
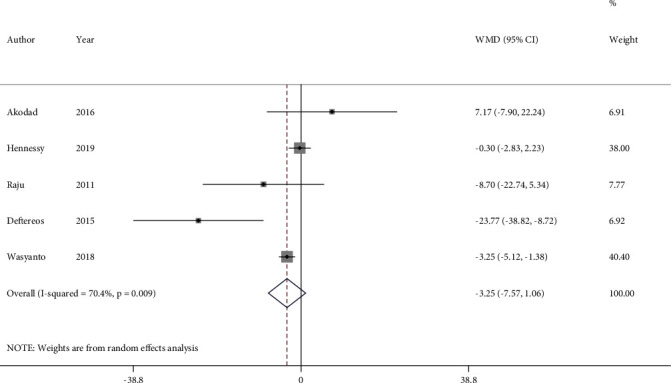
Forest plot displaying weighted mean difference and 95% confidence intervals for the impact of colchicine on high-sensitivity C-reactive protein (hs-CRP) serum level in patients with acute coronary syndrome. WMD: weighted mean difference; CI: confidence interval.

From 6 trials that measured the hs-CRP serum level following colchicine administration, one study was excluded from meta-analysis since it reported geometric mean. Consequently, 5 studies with 532 participants were included in the analysis. Pooled analysis of studies indicated no significant effect on the hs-CRP serum level (WMD = −3.25 mg/L, 95%CI = −7.57 to 1.06) with evidence of high heterogeneity (*I*^2^ = 70.4%, *P* = 0.009) (Figure 2). The effect of colchicine on serum hs-CRP reduction remained nonsignificant after excluding the result of the open-label study (WMD = −3.99 mg/L, 95%CI = −8.41 to 0.43, *I*^2^ = 74.9%, *P* = 0.008) (Figure S1 in the Supplementary Appendix). The intriguing result was observed when we applied standardized mean difference (SMD); colchicine significantly diminished the hs-CRP level with medium power (SMD = −0.41, 95%CI = −0.80 to -0.02, *I*^2^ = 73.4%, *P* = 0.010) (Figure S2 in the Supplementary Appendix).

The subgroup analysis by colchicine dosages revealed that 1 mg colchicine administration per day was associated with significant reduction in the serum hs-CRP level (WMD = −15.98, 95%CI = −30.74 to -1.22) with evidence of moderate heterogeneity (*I*^2^ = 51.4%, *P* = 0.151). However, low-dose colchicine (0.5 mg/d) did not show significant effect (WMD = −1.90, 95%CI = −4.78 to 0.98) (Figure S3).

### 3.2. Primary Composite Endpoint

**Figure 3:**
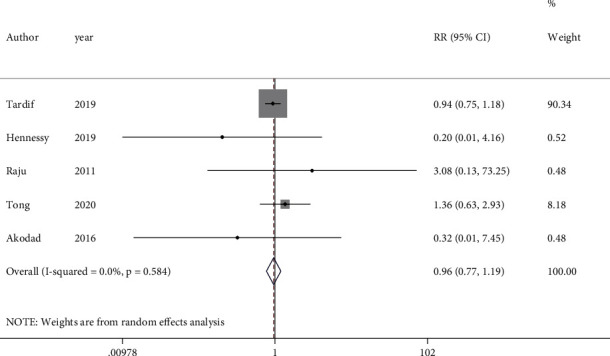
Forest plot displaying risk ratio and 95% confidence intervals for the impact of colchicine on primary composite endpoint in patients with acute coronary syndrome. RR: risk ratio; CI: confidence interval.

From 6 studies which evaluated the all-cause mortality following colchicine administration, one was excluded due to the shorter than one-month duration of follow-up. These studies also evaluated the effect of colchicine on MI recurrence. Therefore, 5 studies with 5895 participants were included in the analysis to assess the composite of death and MI. Results indicated that colchicine consumption had no significant effect on the primary composite endpoint (RR = 0.96, 95%CI = 0.77 to 1.19, *I*^2^ = 0.0%, *P* = 0.584) (Figure 3). The result did not change after conducting sensitivity analysis in the absence of open-label study (RR = 0.96, 95%CI = 0.77 to 1.20, *I*^2^ = 0.0%, *P* = 0.479) (Figure S4). Interestingly, subgroup analysis by the type of diseases indicated inverse direction; while a change toward increase was found in the ACS subgroup, the change in the MI subgroup was reduced (Figure S5).

### 3.3. Myocardial Infarction

**Figure 4:**
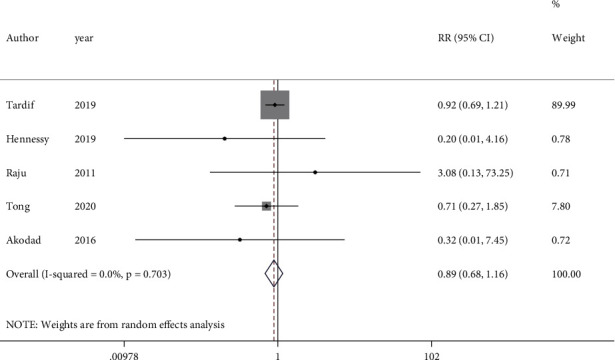
Forest plot displaying risk ratio and 95% confidence intervals for the impact of colchicine on myocardial infarction in patients with acute coronary syndrome. RR: risk ratio; CI: confidence interval.

Five studies with 5859 participants which evaluated the effect of colchicine on MI recurrence were included in the analysis. The RR of MI recurrence did not change following colchicine consumption (RR = 0.89, 95%CI = 0.68 to 1.16, *I*^2^ = 0.0%, *P* = 0.703) (Figure 4). The result remained the same after excluding the open-label studies (RR = 0.89, 95%CI = 0.68 to 1.17, *I*^2^ = 0.0%, *P* = 0.610) (Figure S6).

### 3.4. Stroke

**Figure 5:**
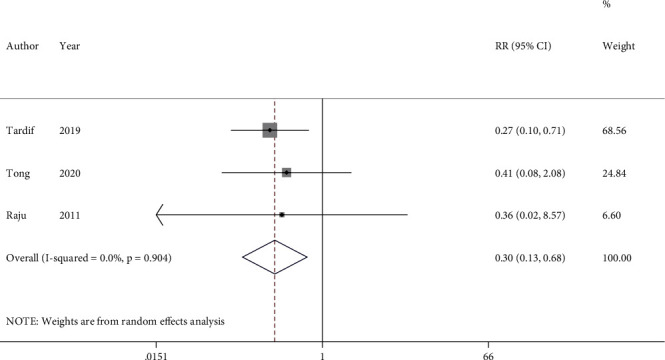
Forest plot displaying risk ratio and 95% confidence intervals for the impact of colchicine on stroke in patients with acute coronary syndrome. RR: risk ratio; CI: confidence interval.

Three studies with 5614 participants which evaluated the effect of colchicine on stroke occurrence were included in the analysis. Pooled analysis of the studies revealed that colchicine usage was significantly associated with a 70% reduction in the risk of stroke (RR = 0.30, 95%CI = 0.13 to 0.68). The heterogeneity among studies was found to be nonsignificant (*I*^2^ = 0.0%, *P* = 0.904) (Figure 5).

### 3.5. Gastrointestinal Adverse Events

**Figure 6:**
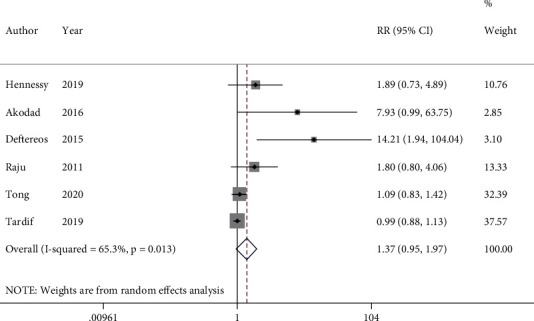
Forest plot displaying risk ratio and 95% confidence intervals for the impact of colchicine on gastrointestinal adverse events in patients with acute coronary syndrome. RR: risk ratio; CI: confidence interval.

The pooled analysis of 6 studies with 5977 participants demonstrated nonsignificantly higher gastrointestinal adverse events in the colchicine group (RR = 1.37, 95%CI = 0.95 to 1.95, *I*^2^ = 65.3%, *P* = 0.013) (Figure 6). Likewise, sensitivity analysis did not reveal a significant difference between the groups (RR = 1.25, 95%CI = 0.90 to 1.74) (Figure S7). Subgroup analysis by colchicine dosage indicated no significant change for 0.5 mg colchicine; however, 1 mg colchicine consumption was associated with about five-fold higher risk of GI adverse events (RR = 4.75, 95%CI = 1.02 to 22.20, *I*^2^ = 64.3%, *P* = 0.061) (Figure S8).

## 4. Discussion

The present analysis evaluated the effect of colchicine as secondary prevention, specifically on ACS and MI. The results of our meta-analysis revealed that ACS and MI patients who had received colchicine had a similar incidence of primary composite endpoint, MI, and GI adverse events when compared with the noncolchicine group. Of note, the stroke occurrence significantly declined following colchicine administration. Another finding was that colchicine revealed a marginally significant effect on the hs-CRP serum level reduction. Inflammation plays a key role in atherosclerosis. This point gives way to the idea of using anti-inflammatory substances as a treatment for coronary heart diseases for instance acute coronary syndrome [22, 23]. Most studies in the field focused only on CAD or evaluated both ACS and CAD together which due to distinct pathophysiology in some aspects (such as acuteness or chronicity) [24] cast doubt on the validity of results.

hs-CRP is a nonspecific inflammatory marker recognized as one of the acute phase reactants which are synthesized and released from the liver [25]. Previous studies have posed predictive effects of the high hs-CRP serum level on adverse clinical outcomes, possibly representative of persistent inflammation [26–29]. Colchicine has pleiotropic inhibitory effects on inflammation including inhibition of microtubule polymerization and also interleukin 1, interleukin 6, and NLRP3 inflammasome activation [30–32]. The key mediator, which controls the synthesis of most acute-phase proteins including hs-CRP, seems to be IL-6 [33]. Therefore, it is theoretically expected and experimentally shown that the colchicine usage decreases the hs-CRP levels which lead to fewer adverse cardiac events. In our current study, although a downward trend was observed for the hs-CRP serum level in the pooled analysis, this effect was nonsignificant. Apart from statistical heterogeneity which was significant for this analysis, methodological heterogeneity among the included studies should gain attention, too. The included studies were different with regard to the type and severity of diseases, loading dose of colchicine, timing of colchicine administration, follow-up duration, and the number of patients undergoing PCI in each study and even different groups of each study. These all can turn to a misleading conclusion. Another explanation for this result is the low number of studies and subjects. Since *P* value closely correlates with the sample size, such that in sufficiently large scale samples almost always significant results are observed [34], considering the effect size for the current study is essential. Interestingly, SMD manifested significant medium-power effect of colchicine on hs-CRP attenuation. The main reason was the implementation of SMD was after omitting akodad et al. study. The mentioned study was not only an open-label but it also measured CRP in the acute phase of ACS (during the index hospitalization) which made us unable to investigate the effect of colchicine on hs-CRP. Other studies measured hs-CRP on the last day of follow-up. The marginally significant effect presented in the sensitivity analysis (Figure S1) by excluding the above-mentioned study could support this explanation. In the subgroup analysis, we found that 1 mg per day colchicine was associated with a significant reduction in hs-CRP. These are molecular promising results which are recommended to be ascertained by clinical outcomes.

The study conducted by Roubille et al. showed the correlation between hs-CRP and infarct size and also that the peak of hs-CRP could be detected within 3 days post-MI [35]. In another study, the time-to-treatment analysis of colchicine initiation on the COLCOT study population revealed that the start of colchicine administration within 3 days was more greatly linked with a favorable composite of hard clinical outcomes [36]. We might be able to speculate that if a substance with a dampening inflammation mechanism is used for atherosclerosis treatment, the optimal outcome might be achieved through early administration. More evidence is needed to support this claim and also to determine the relationship between hs-CRP and hard clinical outcomes. Indeed, based on the current evidence, we are unable to attribute a more favorable outcome in the given study to hs-CRP attenuation as time-to-treatment analysis for the hs-CRP outcome is lacking for this study [12]. An observational study indicated significant hs-CRP reduction and plaque stabilizing effect of low-dose colchicine plus optimal medical treatment in comparison with optimal medical treatment alone. A high linear correlation was found between hs-CRP change and plaque stabilizing effect in this study [19]. Although this point takes us one step closer to linking the molecular results with clinical outcomes, large-scale high-quality randomized controlled trials are needed to confirm it.

The two main hard clinical outcomes which were MI and primary composite endpoint did not change following colchicine administration. Despite the lack of heterogeneity, there are noteworthy points about these analyses. The COLCOT study [12] constituted 90% of the weight of both analyses. In this study, colchicine was started at the median of 13 days after index MI. This, along with the fact that the intervention and control groups were not matched according to culprit lesions or infarct size, could be addressed as confounding factors in the analysis. It was indicated that in the COLCOT the positive effect of colchicine on primary composite endpoint was mostly driven by stroke and urgent hospitalization for angina leading to coronary revascularization which in our study were not counted as components of composite endpoint since they were not reported in most of the studies and the limitations in conducting meta-analysis for such outcomes. Moreover, lack of effect can be partly explained by the study conducted by Tong et al. [20]. Although using colchicine at the time of index hospitalization improved the recurrence of ACS, surprisingly, the number of deaths was significantly higher in the colchicine group. Indeed, 5 out of 8 deaths that occurred in the colchicine group were due to noncardiovascular reasons; 2 of the deceased patients suffered metastatic cancer or acute myeloid leukemia. The other 2 halted colchicine within the first month and sepsis happened 10 months thereafter. Based on the acute nature of sepsis [37], we can barely attribute the occurrence of sepsis in these cases to colchicine consumption.

One unanticipated finding was the inverse directions that were detected for MI and ACS patients regarding the primary composite endpoint. This could suggest that the more acute the inflammatory condition is, the more efficient the colchicine would be. As mentioned above, colchicine has shown better clinical outcomes when applied within the three days post-MI simultaneous with the most acute phase of the inflammation. The last-mentioned finding of our study might emphasize the consistent point, which is colchicine consumption for acute inflammation, in another way. Although the detected trend was statistically nonsignificant in the current study, it is worth evaluating in future research by resolving the limitations of previous studies.

In line with previous studies, in the current study colchicine consumption significantly decreased the occurrence of stroke. Khandkar et al. [38] in their meta-analysis included the studies which reported stroke as an outcome and used colchicine as the intervention. They concluded that colchicine consumption reduced the risk of stroke by about three times. The result was prominently driven by a cohort study that evaluated colchicine as primary prevention in gout patients. The other meta-analysis conducted by Masson et al. [39] focused specifically on the patients with high cardiovascular risk and colchicine as secondary prevention means that they evaluate the effect of colchicine on ACS, CAD, heart failure, and postcardiac surgery and on those who underwent PCI. They reported three-fold fewer strokes in the colchicine group. Likewise, the meta-analysis conducted by Katsanos et al. [40] with a focus on coronary heart disease and also the current study which specifically included ACS patients have demonstrated this promising result. An explanation for the consistency among the last three is that the COLCOT study made about 70% of the weight of each analysis. Due to the restricted number of articles, further studies are needed to elucidate both the primary and secondary preventive effects of colchicine on stroke. Moreover, the mechanism of the antistroke effect of colchicine needs to be determined as in the present study colchicine decreased the risk of stroke contrary to MI while due to similar pathophysiology (which is inflammation), we expected the same result for both. This might be suggestive of a yet unknown colchicine mechanism of action. Improvement of stroke could not be attributed to the hs-CRP reduction in our study due to insufficient information about 2 studies which constituted more than 90% of the weight of the analysis performed for stroke.

As colchicine has a narrow therapeutic window, another subject that should obtain consideration is the adverse effects of this drug. The toxic effect of colchicine on cells has been claimed to be prominently exerted by antimitotic activity. Therefore, the GI tract, skin, hair, and bone marrow with high proliferative activity are at greater risk [41]. Consistent with this point, there is strong evidence that the most common adverse effects are gastrointestinal, such as diarrhea, nausea, and vomiting [30, 42]. Our study revealed similar GI adverse events between the colchicine and non-colchicine groups. Besides, we found that colchicine dosage was associated with GI adverse events and the risk of GI events could be avoided by low-dose (0.5 mg/d) administration. It should be noted that our results should be treated with caution due to high statistical heterogeneity. A meta-analysis involving 14188 cardiovascular patients found that administration of colchicine was associated with nearly a two-fold rise in GI adverse events that could be avoided by low-dose application of colchicine [43]. In addition, Ullah et al. [44] in their meta-analysis reported the significantly greater harmful effect of colchicine on CAD patients. To sum up, while the current research did not indicate a higher risk of GI-related side effects, additional trials with long-term follow-up are certainly required regarding the opposite view of high-quality studies with greater number of participants. Besides, other than GI, there are more extreme side effects that need more attention, such as sepsis and myelotoxicity. Sufficient evidence does not exist to conduct meta-analysis. In future long-term studies, these should be further elucidated, too.

Although the current study focused specifically on ACS patients for more reliable outcomes, there are several limitations to acknowledge. First, it is possible that the methodological heterogeneity among the included studies has affected our results. For instance, the colchicine onset, duration, daily dosage, and loading dose altogether were not completely the same in any of the two included studies. Second, hard clinical outcomes were mostly driven by the COLCOT study as this study made about 70% or more of the weight of these analyses. Indeed, COLCOT is the only multinational large-scale study on this topic. Third, we were unable to conduct a meta-analysis for some clinical outcomes as they were evaluated in only one or two studies. These include atrial fibrillation, hospitalization leading to revascularization, unstable angina, deep vein thrombosis, and pulmonary embolism. It is possible that considering these outcomes as components of MACE would change the result of the present study. This study with all its limitations would provide a basis for precisely designed research in the future along with indicating some possible promising effect of colchicine on ACS. There is still a way to go until allowance of colchicine consumption in daily clinical practice.

## 5. Conclusion

New strategies for the treatment and prevention of coronary heart disease may be identified with the understanding that inflammation plays a pathological function in atherosclerosis. Colchicine appears to have some benefits on stroke. MI and primary composite endpoint did not change following colchicine consumption in our study. Nevertheless, a better result might be achieved by consumption in more acute inflammatory conditions; it means colchicine application for more acute conditions like MI rather than all ACS which includes unstable angina and also consumption as immediate as possible following index MI. Moreover, colchicine revealed marginally significant efficacy for lowering CRP level, which is a prognostic factor for cardiovascular complications. Colchicine, especially in low doses, seems to be relatively safe in the clinical setting. However, this result should be treated with more caution, due to high statistical and methodological heterogeneity and inconsistency with the results of a larger meta-analysis.

## Figures and Tables

**Table 1:** Summary of the PICOS criteria used to identify relevant studies.

Parameter	Description
Population	ACS or MI patients of any age
Intervention	Colchicine
Comparator	Placebo or control group
Outcomes	Primary composite endpoint (MI and death), MI, stroke, hs-CRP serum level, GI adverse events
Study design	Randomized and nonrandomized clinical trials

ACS: acute coronary syndrome; MI: myocardial infarction; hs-CRP: high-sensitivity C-reactive protein; GI: gastrointestinal.

**Table 2:** An overview of clinical trials investigating the effect of colchicine on acute coronary syndrome (ACS) or myocardial infarction (MI).

Author	Year	Country	Study design	Sex	Participants' disease	Colchicine dosage	Control	Age, years (intervention/control)	Duration of colchicine usage	Duration of follow-up	Sample size, *N* (intervention/control)	Endpoint (s)
Mariama Akodad [32]	2016	France	Clinical trial	Both	STEMI	Colchicine 1 mg daily+optimal medical care	Optimal medical care alone	60.1/59.7	1 month	1 month	23/21	CRP, all-cause mortality, GI adverse events
Thomas Hennessy [33]	2019	Australia	RCT	Both	Acute MI	0.5 mg daily	Placebo	61/61	1 month	1 month	119/118	hs-CRP, all-cause mortality, MI, GI adverse events
Nina C. Raju [34]	2011	Australia	RCT	Both	ACS or ischemic stroke	1 mg daily	Placebo	57.2/57.2	1 month	1 month	36/38	Hs-CRP, all-cause mortality, stroke, MI, GI adverse events
Spyridon Deftereos [35]	2015	Greece	RCT	Both	STEMI	Loading dose of 2 mg continuing with 0.5 mg bid (if body weight < 60 kg then 0.5 mg daily)	Placebo	58/58	5 days	5 days	77/74	hs-CRP, GI adverse events
Jean-Claude Tardif [36]	2019	Multinational	RCT	Both	MI	0.5 mg daily	Placebo	62.1/61.2	19.6 and 19.5 months in colchicine and placebo groups, respectively (median)	Median of 22.6 months overall and 6 months in hs-CRP substudy	2366/2379 overall and 99/108 in hs-CRP substudy	All-cause mortality, stroke, MI, GI adverse events
Trisulo Wasyanto [12]	2018	Indonesia	RCT	Both	Acute MI	0.5 mg daily	Placebo	57.8/52.8	5 days	5 days	16/16	hs-CRP
David C. Tong [19]	2020	Australia	RCT	Both	ACS	0.5 mg colchicine bid for the first month, followed by 0.5 mg daily for eleven months	Placebo	59.7/60.0	12 months	365 days	396/399	All-cause mortality, stroke, MI, GI adverse events

RCT: randomized controlled trial; ACS: acute coronary syndrome; MI: myocardial infarction; STEMI: ST-Elevation Myocardial Infarction; hs-CRP: high-sensitivity C-reactive protein; GI: gastrointestinal.

**Table 3:** Summary of methodological quality assessment of the included studies.

Study	Random sequence generation (selection bias)	Allocation concealment (selection bias)	Blinding of participants and personnel (performance bias)	Blinding of outcome assessment (detection bias)	Incomplete outcome data (attention bias)	Selective reporting (reporting bias)
Jean-Claude Tardif	U	U	L	L	L	L
David C. Tong	L	L	L	L	L	L
Thomas Hennessy	U	L	L	L	L	L
Nina C. Raju	L	L	L	L	L	L
Spyridon Deftereos	L	L	L	L	L	L
Mariama Akodad	U	U	H	H	L	L
Trisulo Wasyanto	U	U	U	U	L	L

H: high risk of bias; L: low risk of bias; U: unclear.

## Data Availability

Data would be available pending the request from corresponding author.

## Supplementary Materials

Supplementary Materials Figure S1: forest plot displaying weighted mean difference and 95% confidence intervals for the impact of colchicine on high-sensitivity C-reactive protein (hs-CRP) serum level in patients with acute coronary syndrome and sensitivity analysis by omitting the result of open-label study. WMD: weighted mean difference; CI: confidence interval. Figure S2: forest plot displaying standardized mean difference and 95% confidence intervals for the impact of colchicine on high-sensitivity C-reactive protein (hs-CRP) serum level in patients with acute coronary syndrome. SMD: standardized mean difference; CI: confidence interval. Figure S3: forest plot displaying weighted mean difference and 95% confidence intervals for the impact of colchicine on high-sensitivity C-reactive protein (hs-CRP) serum level in patients with acute coronary syndrome, subgroup analysis according to colchicine dosages. WMD: weighted mean difference; CI: confidence interval. Figure S4: forest plot displaying risk ratio and 95% confidence intervals for the impact of colchicine on primary composite endpoint in patients with acute coronary syndrome and sensitivity analysis by omitting the result of open-label study. RR: risk ratio; CI: confidence interval. Figure S5: forest plot displaying risk ratio and 95% confidence intervals for the impact of colchicine on primary composite endpoint in patients with acute coronary syndrome and subgroup analysis according to type of diseases. RR: risk ratio; CI: confidence interval. Figure S6: forest plot displaying risk ratio and 95% confidence intervals for the impact of colchicine on myocardial infarction in patients with acute coronary syndrome and sensitivity analysis by omitting the result of open-label study. RR: risk ratio; CI: confidence interval. Figure S7: forest plot displaying risk ratio and 95% confidence intervals for the impact of colchicine on gastrointestinal adverse events in patients with acute coronary syndrome and sensitivity analysis by omitting the result of open-label study. RR: risk ratio; CI: confidence interval. Figure S8: forest plot displaying risk ratio and 95% confidence intervals for the impact of colchicine on gastrointestinal adverse events in patients with acute coronary syndrome and subgroup analysis according to colchicine dosages. RR: risk ratio; CI: confidence interval.
