# Supplementary material for: Effect of Treatment with Colchicine after Acute Coronary Syndrome on Major Cardiovascular Events: A Systematic Review and Meta-Analysis of Clinical Trials
Source: Cardiovasc Ther. 2022 Apr 13;2022:8317011. doi: 10.1155/2022/8317011 (PMC9020977; doi:10.1155/2022/8317011)
Supplement: Supplementary Materials — Figure S1: forest plot displaying weighted mean difference and 95% confidence intervals for the impact of colchicine on high-sensitivity C-reactive protein (hs-CRP) serum level in patients with acute coronary syndrome and sensitivity analysis by omitting the result of open-label study. WMD: weighted mean difference; CI: confidence interval. Figure S2: forest plot displaying standardized mean difference and 95% confidence intervals for the impact of colchicine on high-sensitivity C-reactive protein (hs-CRP) serum level in patients with acute coronary syndrome. SMD: standardized mean difference; CI: confidence interval. Figure S3: forest plot displaying weighted mean difference and 95% confidence intervals for the impact of colchicine on high-sensitivity C-reactive protein (hs-CRP) serum level in patients with acute coronary syndrome, subgroup analysis according to colchicine dosages. WMD: weighted mean difference; CI: confidence interval. Figure S4: forest plot displaying risk ratio and 95% confidence intervals for the impact of colchicine on primary composite endpoint in patients with acute coronary syndrome and sensitivity analysis by omitting the result of open-label study. RR: risk ratio; CI: confidence interval. Figure S5: forest plot displaying risk ratio and 95% confidence intervals for the impact of colchicine on primary composite endpoint in patients with acute coronary syndrome and subgroup analysis according to type of diseases. RR: risk ratio; CI: confidence interval. Figure S6: forest plot displaying risk ratio and 95% confidence intervals for the impact of colchicine on myocardial infarction in patients with acute coronary syndrome and sensitivity analysis by omitting the result of open-label study. RR: risk ratio; CI: confidence interval. Figure S7: forest plot displaying risk ratio and 95% confidence intervals for the impact of colchicine on gastrointestinal adverse events in patients with acute coronary syndrome and sensitivity analysis by omitting t [file 8317011.f1.docx]

**Supplementary materials**

**Figure S1.** Forest plot displaying weighted mean difference and 95% confidence intervals for the impact of colchicine on high-sensitivity C-reactive Protein (hs-CRP) serum level in patients with acute coronary syndrome, sensitivity analysis by omitting the result of open-label study. WMD: weighted mean difference; CI: Confidence interval.

**Figure S2.** Forest plot displaying standardized mean difference and 95% confidence intervals for the impact of colchicine on high-sensitivity C-reactive Protein (hs-CRP) serum level in patients with acute coronary syndrome. SMD: standardized mean difference; CI: Confidence interval.

**Figure S3.** Forest plot displaying weighted mean difference and 95% confidence intervals for the impact of colchicine on high-sensitivity C-reactive Protein (hs-CRP) serum level in patients with acute coronary syndrome, subgroup analysis according to colchicine dosages. WMD: weighted mean difference; CI: Confidence interval.

**Figure S4.** Forest plot displaying risk ratio and 95% confidence intervals for the impact of colchicine on primary composite endpoint in patients with acute coronary syndrome, sensitivity analysis by omitting the result of open-label study. RR: risk ratio; CI: Confidence interval.

**Figure S5.** Forest plot displaying risk ratio and 95% confidence intervals for the impact of colchicine on primary composite endpoint in patients with acute coronary syndrome, subgroup analysis according to type of diseases. RR: risk ratio; CI: Confidence interval.

**Figure S6.** Forest plot displaying risk ratio and 95% confidence intervals for the impact of colchicine on myocardial infarction in patients with acute coronary syndrome, sensitivity analysis by omitting the result of open-label study. RR: risk ratio; CI: Confidence interval.

**Figure S7.** Forest plot displaying risk ratio and 95% confidence intervals for the impact of colchicine on gastrointestinal adverse events in patients with acute coronary syndrome, sensitivity analysis by omitting the result of open-label study. RR: risk ratio; CI: Confidence interval.

**Figure S8.** Forest plot displaying risk ratio and 95% confidence intervals for the impact of colchicine on gastrointestinal adverse events in patients with acute coronary syndrome, subgroup analysis according to colchicine dosages. RR: risk ratio; CI: Confidence interval.
